# Rational Design of a New *Trypanosoma rangeli* Trans-Sialidase for Efficient Sialylation of Glycans

**DOI:** 10.1371/journal.pone.0083902

**Published:** 2014-01-03

**Authors:** Carsten Jers, Malwina Michalak, Dorte M. Larsen, Kasper P. Kepp, Haiying Li, Yao Guo, Finn Kirpekar, Anne S. Meyer, Jørn D. Mikkelsen

**Affiliations:** 1 Center for BioProcess Engineering, Department of Chemical and Biochemical Engineering, Technical University of Denmark, Lyngby, Denmark; 2 Department of Chemistry, Technical University of Denmark, Lyngby, Denmark; 3 Department of Biochemistry and Molecular Biology, Southern University of Denmark, Odense, Denmark; University of Houston, United States of America

## Abstract

This paper reports rational engineering of *Trypanosoma rangeli* sialidase to develop an effective enzyme for a potentially important type of reactivity: production of sialylated prebiotic glycans. The *Trypanosoma cruzi* trans-sialidase and the homologous *T. rangeli* sialidase has previously been used to investigate the structural requirements for trans-sialidase activity. We observed that the *T. cruzi* trans-sialidase has a seven-amino-acid motif (197–203) at the border of the substrate binding cleft. The motif differs substantially in chemical properties and substitution probability from the homologous sialidase, and we hypothesised that this motif is important for trans-sialidase activity. The 197–203 motif is strongly positively charged with a marked change in hydrogen bond donor capacity as compared to the sialidase. To investigate the role of this motif, we expressed and characterised a *T. rangeli* sialidase mutant, Tr13. Conditions for efficient trans-sialylation were determined, and Tr13's acceptor specificity demonstrated promiscuity with respect to the acceptor molecule enabling sialylation of glycans containing terminal galactose and glucose and even monomers of glucose and fucose. Sialic acid is important in association with human milk oligosaccharides, and Tr13 was shown to sialylate a number of established and potential prebiotics. Initial evaluation of prebiotic potential using pure cultures demonstrated, albeit not selectively, growth of Bifidobacteria. Since the 197–203 motif stands out in the native trans-sialidase, is markedly different from the wild-type sialidase compared to previous mutants, and is shown here to confer efficient and broad trans-sialidase activity, we suggest that this motif can serve as a framework for future optimization of trans-sialylation towards prebiotic production.

## Introduction

For production of human milk oligosaccharides (HMOs), glycan sialylation can be achieved chemically as well as enzymatically [1]. To achieve enzymatic synthesis, a trans-sialidase (TcTS) derived from *T. cruzi*, the causative agent of Chagas disease, has previously proven useful by transferring sialic acid from a donor to an acceptor glycan [2]. However, for industrial production of food-grade HMOs, it is a drawback that the enzyme constitutes an important virulence factor within *T. cruzi* [3]. Redesigning mutants of the non-pathogenic *T. rangeli* sialidase (TrSA) that possesses relatively low trans-sialidase activity [4] provides an attractive alternative for application in bioconversion processes. TrSA is 70% identical to TcTS and has the same overall tertiary structure. Both enzymes have been extensively characterised by biochemical, mutational and structural studies, and they share a common double displacement mechanism with a tyrosine as the catalytic nucleophile [5], [6].

In TcTS, the acceptor binding site consists of Y119 and W312 which form stacking interactions with the acceptor sugar [7]. In TrSA, W313 (corresponds to W312 in TcTS) is found in a different conformation due to a Q284P substitution, while the Y120 (corresponds to Y119 in TcTS) is replaced by serine [8]. In addition to these differences in the acceptor binding site, a conserved D97 hydrogen bonds differently to sialic acid in the two enzymes, possibly due to the substitutions V96M and P98A. Correction of both the acceptor-binding site (S120Y, G249Y, and Q284P) and the sialic acid binding pocket (M96V, and A98P) is required to confer trans-sialidase activity (1% of TcTS activity) to TrSA, and the additional single mutations I37L (in this study named Tr6) and G342A further increase activity to 10% of the TcTS activity [9], [4]. Kinetic data, however, indicate that the mutants display a >25-fold lower affinity for lactose and >100-fold higher *k*_cat_ for the undesired, competing hydrolysis [4], indicating ample room for further improvement.

In this study, we carried out a rational design of the *T. rangeli* mutant sialidase Tr6 based on the identification of a major difference (the motif constituted by amino acids 197–203) close to the binding cleft. We hypothesised that this motif might affect the trans-sialidase *vs*. hydrolase activity, notably due to the extremely unusual (and substitution-wise unlikely) +3 charge change in this segment of the native trans-sialidase. Our production and characterisation of a *T. rangeli* mutant sialidase (Tr13) from the Tr6 parent incorporating this motif confirmed this hypothesis, since a reduced hydrolytic activity was observed which promoted overall trans-sialylation efficiency. Using a side-stream component from the dairy industry, casein glycomacropeptide (cGMP), as sialic acid donor for Tr13, we were able to sialylate a number of established and potential prebiotic glycans, demonstrating a broad acceptor specificity of Tr13.

## Materials and Methods

### Substrates

*para*-Nitrophenyl neuraminic acid (pNP-Neu5Ac) was purchased from Sigma-Aldrich (Steinheim, Germany). 3′-sialyllactose and 6′-sialyllactose were obtained from Carbosynth (Compton, United Kingdom). The commercial casein glycomacropeptide (cGMP) product LACPRODAN CGMP-20 with a sialic acid content of about 9% (w/w) was supplied by Arla Foods (Viby, Denmark), and low molecular weight impurities were removed by filtration with a 5 kDa membrane. 4-methylumbelliferyl-β-D-galactopyranoside (MU-Gal), β-lactose, lactulose, melibiose, maltose, and fucose were purchased from Sigma-Aldrich (Steinheim, Germany). Galactooligosacchardies (GOS) were purchased from Gulio Gross (Trezzano, Italy). Iso-maltooligosaccharides (IMO) were kindly provided by Taka Hayashi from Kyoto University, RISH (Gokasho, Japan).

### Rational mutant selection

Sialidase catalytic domains were identified using NCBI Conserved Domain Search [10]. Pymol v1.3 (Schrödinger) was used to identify amino acids within 14 Å of the sialic acid binding site, based on the crystal structure with PDB code 1WCS, and was also used for preparing the figures. The *T. rangeli* sialidase mutant Tr6 (see below) and trans-sialidases from *T. cruzi* (TcTS) (Uniprot ID Q26966), *Trypanosoma congolense* (Uniprot ID G0WJG3) [11] and *Trypanosoma brucei* (Uniprot ID Q57XJ2) [12] were aligned using ClustalW [13]. Ranking of chemical difference between substituted amino acids in Tr6 *vs*. TcTS was done based on being first- or second sphere relative to the substrate and based on the polar/nonpolar and small/large distinction; such property-based selection turned out to correlate well with standard substitution matrices (BLOSUM62), i.e. the most unlikely substitutions were considered noteworthy. A comparison of loop structures in *T. cruzi* and *T. rangeli* with and without a substrate analogue DANA (2, 3-Dehydro-2-deoxy-N-acetylneuraminic acid) bound was made using structures of TcTS (PDB codes 1MS1 and 1MS3) and TrSA (PDB codes 1N1S and 1N1T). A 3D-model of Tr13 was made using the Modeller software [14] with 1WCS as the template.

### DNA manipulations and strain construction

A gene encoding Tr6, *T. rangeli* sialidase mutant TrSA_5mut_ (the sequence was extracted from PDB file 1WCS) with an additional mutation I37L [4], was codon-optimized for expression in *Pichia pastoris* and synthesized by DNA2.0 (Menlo Park, CA, USA). The synthesized gene was inserted in the *P. pastoris* expression vector pPICZαC (Invitrogen) between the *Xho*I and *Xba*I restriction sites generating a translational fusion to the α-factor signal sequence as well as a C-terminal *c-myc* epitope and a 6xHis tag (Figure S1). The plasmid was propagated in *Escherichia coli* NM522 grown at 37°C with shaking in low salt LB medium (10 g/L tryptone, 5 g/L yeast extract, and 5 g/L NaCl) supplemented with 25 µg/mL zeocin. This vector, pPICZα-Tr6, was used as a template for introduction of additional mutations by PCR using overlapping primers (Table 1). The PCR products were inserted in pPICZαC between the *Xho*I and *Xba*I sites. Constructs were sequenced to confirm the mutations and to assure that no unwanted mutations had been introduced by PCR. In the following, the mutants are denoted by the amino acid change compared to the parent (e.g. Tr6 Q123R), except for the multi-mutant denoted Tr13, in which amino acids 197–203 were changed from IADMGGR to VTNKKKQ. *P. pastoris* X-33 was transformed with the constructs by electroporation following the manufacturer's instructions (Invitrogen).

**Table 1:** List of primers.

Name	Sequence	Description
**Tr_fwd**	GCTCTCGAGAAGAGAGAGGCTGAAG	*Xho*I, Tr 5′
**Tr_rev**	CGCTCTAGAAATGCTGCTGTACCAGC	*Xba*I, Tr 3′
**Q123S_F**	CTATTGGACC**TCT**CACAGAGATGGATCTGACTGG	Q123S
**Q123S_R**	CATCTCTGTG**AGA**GGTCCAATAGTTCCTTGTCTTG	Q123S
**R125G_F**	GACCCAGCAC**GGA**GATGGATCTGACTGGGAACC	R125G
**R125G_R**	CAGATCCATC**TCC**GTGCTGGGTCCAATAGTTCC	R125G
**G127A_F**	GCACAGAGAT**GCT**TCTGACTGGGAACCATTGTTG	G127A
**G127A_R**	CCCAGTCAGA**AGC**ATCTCTGTGCTGGGTCCAATAG	G127A
**E175Q_F**	ACTTACTAAG**CAG**TTCGTAGGTGGAGTAGGCG	E175Q
**E175Q_R**	CTCCACCTACGAA**CTG**CTTAGTAAGTATGCCGTCGAACTC	E175Q
**V177L_F**	TAAGGAATTC**TTG**GGTGGAGTAGGCGCCG	V177L
**V177L_R**	CCTACTCCACC**CAA**GAATTCCTTAGTAAGTATGCCGTCG	V177L
**V180A_F**	CGTAGGTGGA**GCT**GGCGCCGCCATCGTG	V180A
**V180A_R**	TGGCGGCGCC**AGC**TCCACCTACGAATTCCTTAGTAAG	V180A
**G202K_F**	TGCTGACATG**AAG**GGAAGAGTATTTACAAAAATTATGTATTCC	G202K
**G202K_R**	ATACTCTTCC**CTT**CATGTCAGCAATTTGCACAG	G202K
**N250R_F**	AGTCGATTAC**AGA**AGACGTCTGGTGTACGAATCC	N250R
**N250R_R**	CCAGACGTCT**TCT**GTAATCGACTCGGTTATTAATGATTAGC	N250R
**D363E_F**	GAGATTAATACTAAT**GAG**GTTTATTCTCTTGTTTTTGTCCG	D363E
**D363E_R**	CAAGAGAATAAAC**CTC**ATTAGTATTAATCTCATGTAGGGAATATAATTTATC	D363E
**13MUT_F**	CCCTGTGCAA**GTAACTAATAAGAAGAAGCAA**GTATTTACAAAAATTATGTATTCCGAGG	13MUT
**13MUT_R**	TTGTAAATAC**TTGCTTCTTCTTATTAGTTAC**TTGCACAGGGTATACCAAATTAC	13MUT
**P98A_F**	GGTTGTCGAT**GCT**ACGGTCATAGTAAAGGGAAATAAGTTG	P98A
**P98A_R**	CTATGACCGT**AGC**ATCGACAACCCTTGAAACTG	P98A
**Y249G_F**	CCGAGTCGAT**GGA**AATAGACGTCTGGTGTACGAATC	Y249G
**Y249G_R**	GACGTCTATT**TCC**ATCGACTCGGTTATTAATGATTAGC	Y249G

Restriction sites are underlined and mutated nucleotides in bold.

### Protein synthesis and purification

For small-scale protein synthesis, *P. pastoris* X-33 strains harboring pPICZα with mutated genes were grown for three days in 180 mL BMMY (10 g/L yeast extract, 20 g/L peptone, 100 mM potassium phosphate (pH 6), 13.4 g/L yeast nitrogen base, 0.4 mg/L biotin and 0.5% methanol) shaking at 30°C. Protein synthesis was induced every 24 hours by addition of methanol to a final concentration of 0.5%. Cells were removed by centrifugation for 5 min at 3000 g and the supernatant was subsequently sterile filtered using a 0.2 µM Minisart filter (Sartorius AG). The supernatant was concentrated about 100-fold using Vivaspin20 concentrators with a 30 kDa cutoff (Sartorius AG). The 6xHis-tagged protein was purified from concentrated samples using Ni-sepharose (GE Healthcare) columns in accordance with manufacturer's instructions, followed by desalting on PD-10 columns (GE Healthcare) using 20 mM sodium phosphate buffer (pH 7.4), containing100 mM NaCl, and 10% glycerol. The protein sample was finally concentrated to about 200 µL using a Vivaspin0.5 concentrator with a 50 kDa cutoff (Sartorius AG).

For large-scale production, Tr6, Tr13, and Tr6 D363E were produced in a 5 L Sartorius Biostat Aplus fermentor as described previously [15]. The enzymes were purified by Cu^2+^ affinity column chromatography as described previously [16]. Protein concentrations were determined using the BCA protein assay (Thermo Scientific) with bovine serum albumin as standard.

### Sialidase activity assays

Sialidase activity was measured in a reaction containing 50 mM phosphate-citrate buffer (pH 7), 0.75 mM pNP-NeuAc, and 3 µg/mL sialidase enzyme. The reactions were initiated by addition of substrate and were then followed spectrophotometrically at 410 nm at 30°C. pH 7 was chosen to enable detection of released pNP in a continuous assay. Reaction rates were normalized as percent of the activity of Tr6. For measurement of hydrolysis of natural substrates, the assay was done with either 1 mM 3′-sialyllactose, 1 mM 6′-sialyllactose, or 1 mM cGMP-bound sialic acid in 50 mM phosphate-citrate buffer (pH 5) using 1 µg/mL enzyme. Reactions were started by addition of enzyme and stopped by adding H_2_SO_4_ to a final concentration of 45 mM. Quantification of free sialic acid was done using a 2-thiobarbituric acid assay [17] with the modification that butanol extraction was substituted with mixing with dimethyl sulfoxide (DMSO) [18].

### Trans–sialidase activity assay

Trans-sialidase activity was assayed as described previously [19] but with several modifications. Reactions were done in 50 mM phosphate-citrate (pH 6) at 30°C using 2.9 µg/mL enzyme. As donor substrate, 1 mM cGMP-bound sialic acid was used, and MU-Gal was used as acceptor. The low solubility in aqueous solution prevented the use of higher concentrations of MU-Gal than 0.5 mM. A solution of 87 mM MU-Gal in DMSO was diluted to 2 mM in 50 mM phosphate-citrate buffer (pH 6) immediately before preparing the reactions. When crude enzyme preparations from *P. pastoris* were used, a background signal likely related to cleavage of MU-Gal by endogenous β-galactosidase was present. This background signal could be removed by washing the column eight times with 440 µL of 5 mM HCl after sample application without desorbtion of the sialylated product and this was therefore done routinely.

### Optimisation of conditions for trans-sialylation

To determine the optimal conditions (pH, temperature, and concentration of donor and acceptor), a quadratic central composite design was used. MODDE Version 7.0.0.1 (Umetrics AB, Umeå, Sweden) was used as a tool to design the experimental frame and to fit and analyse the data by multiple linear regression analysis. The pH regimes 3, 4 and 5, incubation temperatures 20, 40 and 60°C, and concentrations of the acceptor lactose of 117, 234 and 351 mM were tested. Reactions were done using a fixed concentration of cGMP-bound sialic acid of 8 mM in 15 mM phosphate-citrate buffer with specified pH values, and using 15 µg/mL of Tr13 enzyme. Lactose and cGMP were solubilised in buffer and preincubated at specific temperatures, before the reactions were initiated by addition of enzyme. The biocatalysis process was allowed to proceed for 20 min before the reaction was stopped by heating for 10 min at 90°C. The concentration of sialyllactose was determined by HPAEC as described below.

For a time study of the trans-sialylation reaction, Tr13 was incubated as described above at pH 3 with 351 mM lactose, 8 mM cGMP-bound sialic acid and a reaction temperature of 25°C. The reaction was followed by sampling over a 100 min period, and the concentration of sialyllactose was determined by LC/MS as described below. Measurement at zero time was done using heat-inactivated enzyme. Three replicates were made and each data series fitted to a second-order polynomial function. For each data series, the slope at time zero was used to calculate the specific activity and the standard deviation.

### Enzymatic production and purification of sialylated glycans

The reactions were carried out in stirred glass bottles in reaction volumes of 50 mL for melibiose and maltose, 88 mL for fucose, 100 mL for lactulose, and 250 mL for GOS and IMO. The reaction was performed in 15 mM phosphate-citrate buffer (pH 3) with 351 mM sialic acid acceptor (GOS, IMO, lactulose, melibiose, maltose and fucose) and 8 mM cGMP-bound sialic acid at 25°C using 15 µg/mL enzyme. Prior to the reaction, the substrates were pre-incubated in the buffer. The reaction was carried out for 20 minutes and then stopped by enzyme inactivation by heating at 90°C for 10 minutes.

The reaction mixture was applied to a HiScale 50/20 (GE Healthcare) anion exchange chromatography column packed with 402 mL of Sepharose Q FF. The separation was done at ambient temperature with an ÄKTA purifier 100 work station equipped with a P-900 pump, UV-900 monitor, and Frac-950 fraction collector, all controlled by UNICORN software (GE Healthcare). The elution was monitored at 210 nm. Elution was performed at a flow rate of 70 mL/min. Before injection, the column was equilibrated with 5 column volumes (CV) of water. After injection, the column was washed with 3 CV of water, followed by elution with 3.5 CV of 40 mM ammonium formate and subsequently with 2 CV of 400 mM ammonium formate for cleaning the column. After elution, the column was regenerated with 3 CV of water. Fractions of interest were collected automatically. The products were lyophilized and ammonium formate was removed by repeated solubilization and lyophilization. Product structures were determined by LC/MS, as described below.

### High-performance anion exchange chromatography (HPAEC-PAD)

Quantification of sialyllactose was carried out by HPAEC-PAD analysis using a Dionex BioLC system consisting of GS50 gradient pumps, ED50 electrochemical detector, AS50 chromatography compartment coupled to an AS50 autosampler (Dionex Corp., Sunnyvale, CA). Samples (10 µL) were injected on a CarboPac™ PA1 (4 mm×250 mm) analytical column (Dionex Corp., Sunnyvale, CA) at a flow rate of 1 mL/min. The elution program was based on the method described in [20] except for the modifications in the eluent system given below. The eluent system comprised of deionised water (A), 0.5 M NaOH (B), 1 M NaOAc (C). For the first 3 min an isocratic elution of 80: 20 (% A:B) was applied, which was followed by a linear gradient from 80∶20 (% A:B) to 60∶20∶20 (% A:B:C) from 3 to 27 min. Strongly retained anions were removed from the column by isocratic elution at 40∶20∶40 (% A:B:C) from 27 to 31 min. Subsequently, the column was re-equilibrated for 7 min with 80∶20 (%A:B).

### Capillary Liquid Chromatography/Mass spectrometry

For liquid chromatography/Mass spectrometry (LC/MS) analysis, an Agilent 1100 LC/Agilent 6340 ion trap MS system was used. Oligosaccharides were separated using a Hypercarb porous graphitic carbon (PGC) column (0.32×150 mm, 5 µm, Thermo scientific) at 30°C. Samples (0.5 µL) were loaded onto the column in 10 mM ammonium bicarbonate. Gradient elution was achieved using a binary solvent system consisting of (A) 10 mM ammonium bicarbonate, adjusted to pH 8.5 with ammonium hydroxide, and (B) 100% acetonitrile at a flow rate of 5 µL/min. The gradient was initially at 98∶2 (% A:B) for 5 min, followed by a linear increase to 42∶58 (% A:B) at 33 min. This concentration of B was held for 3 min. Subsequently the eluent was returned to 98∶2 (% A:B) at 40 min and the system was allowed to equilibrate for 10 min prior to the next injection. All solvents used were of the highest HPLC grade. The mass spectrometry was performed in negative ion mode, and was scanned in the range m/z 150–2200 (2 microscans, maximum accumulation time of 150 ms, an ion current count of 200,000) followed by data-dependent MS2 scans of the four most abundant ions in each MS1 scan.

### Bacterial growth assays on sialylated glycans

For testing growth of bacterial strains on sialylated glycans the following strains were used: *Bifidobacterium longum longum* (Danisco Global Culture Collection DGCC 232), *Bifidobacterium longum infantis* (DGCC 233), *Bifidobacterium longum infantis* (DGCC 1497), *Bifidobacterium longum infantis* (DGCC 2238), *Lactobacillus acidophilus* (NCFM, ATCC 700396), *Bifidobacterium longum* (Bl-05, DGCC 9917), *Bifidobacterium lactis* (HN019, DGCC2013), and *Clostridium perfringens* (ATCC 13124). Galactan from potato (Megazyme International LTD, Bray, Co. Wicklow, Ireland) was used as an established prebiotic standard control. The substrates were dissolved in water at 10% (w/v) and sterilized by sterile filtration (0.2 µm Minisart, Sartorius AG, Göttingen, Germany) except for a control substrate, galactan from potato (Megazyme International LTD, Bray, Co. Wicklow, Ireland), that due to its high viscosity was sterilised by UV-radiation for 30 seconds. The strains were precultured in MRS^−^ medium (de Man, Rogosa and Sharpe medium without glucose) with no additional sugars added for 24 h at 37°C under anaerobic conditions before being diluted with fresh MRS^−^ medium to 1% (v/v). Growth on test substrates was done by adding 20 µL of 10% test substrates and 180 µL 1% cell suspension in multiwell plates and growth was followed by measurement of optical density at 600 nm (OD_600_) using Biolink® software (Labsystems) in a Bioscreen® C system (Labsystems, Helsinki, Finland) as described previously [21]. The growth in MRS^−^ medium without addition of carbohydrates was used as control. The experiments were done in three replicates for each strain, and carbohydrate substrate and growth was determined as the area under the growth curve. Data are given as mean values ± standard error.

One-way analyses of variances (one-way ANOVA): 95% confidence intervals were compared as Tukey-Kramer intervals calculated from pooled standard deviations (Minitab Statistical Software, Addison-Weseley, Reading, MA).

## Results and Discussion

### Rational design of the *T. rangeli* sialidase mutant Tr13

**Figure 1:**
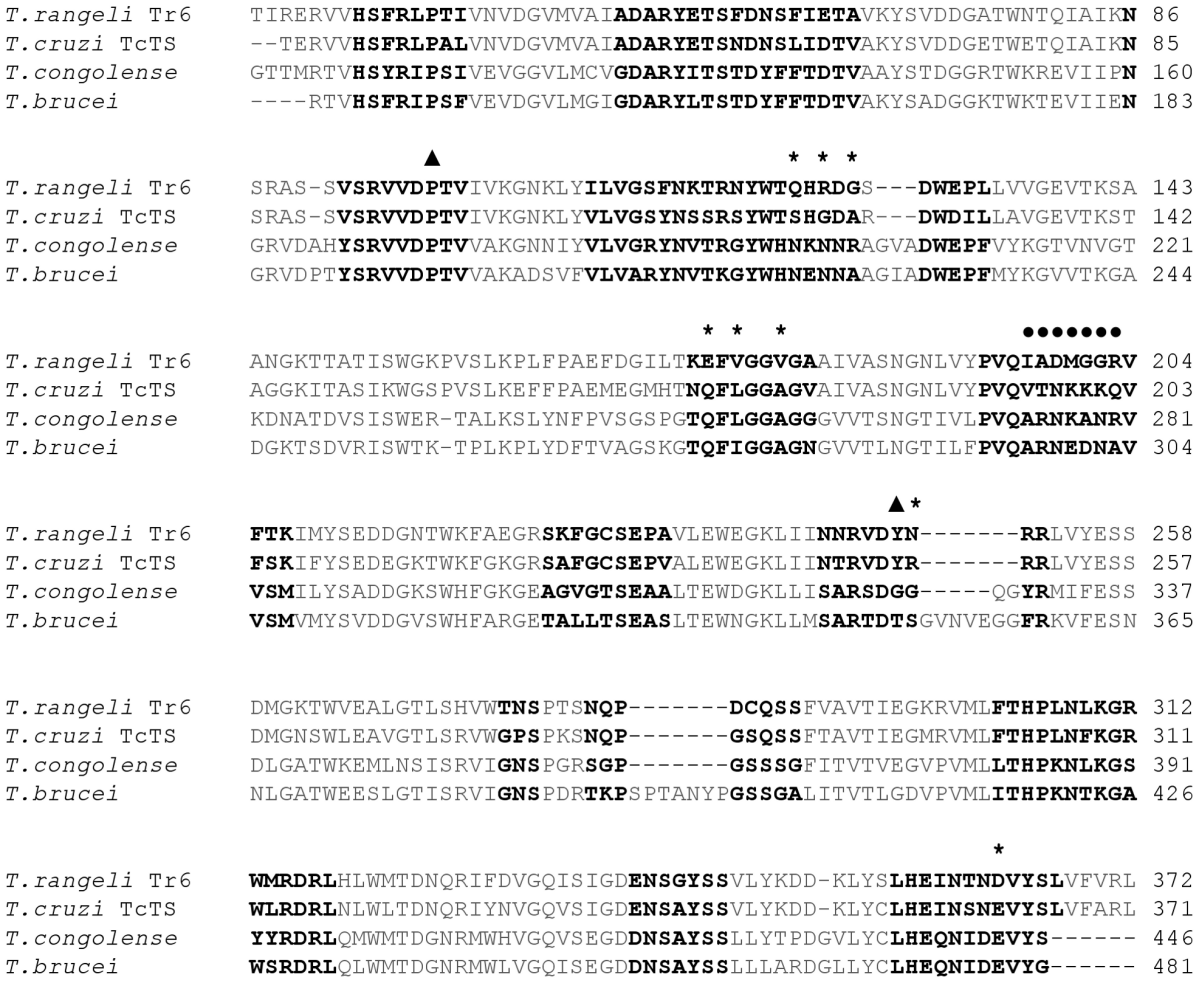
Sequence alignment of sialidase catalytic domain from Tr6 and related trans-sialidases. Tr6 and trans-sialidases from *T. cruzi* (Uniprot ID Q26966), *T. congolense* (Uniprot ID G0WJG3) and *T. brucei* (Uniprot ID Q57XJ2) were aligned using ClustalW. Amino acids within 14 Å of sialic acid binding site are shown in bold. The seven amino acid motif is indicated with filled circles, reverting mutations are indicated with a triangle while other mutated sites are indicated with asterisks.

In order to identify mutations likely to affect enzyme activity, we used two initial criteria: First, we considered only amino acids within 14 Å of the sialic acid-binding site. Secondly, based on an alignment between Tr6 and the efficient trans-sialidase TcTS, we considered the chemical difference between the amino acids, assuming that larger chemical differences, correlating with lower probability of substitution by random evolution, would be the most likely candidates for conferring increased trans-sialidase activity and/or reduce unwanted hydrolysis. We also inspected the impact of changes in surface exposure, hydrogen bonding, and the distance from the acceptor binding site. A sequence alignment with amino acids within 14 Å of the sialic acid-binding site and sites selected for mutagenesis are shown in Figure 1.

**Figure 2:**
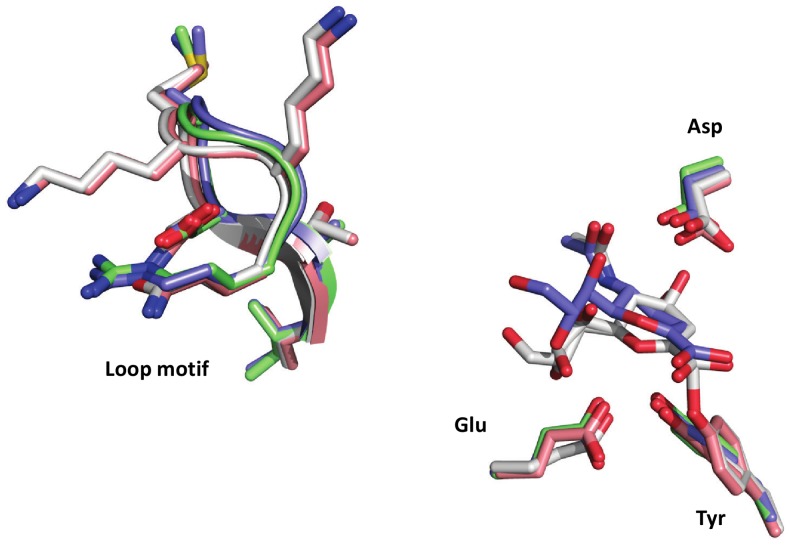
Comparison of the loop motif in TcTS and TrSA. Structures of TcTS (PDB 1MS1; grey and 1MS3; light red) and TrSA (PDB 1N1T; blue and 1N1S; green), with and without the sialic acid analogue DANA bound respectively, were compared. The loop motif constituted by amino acids 196–202/197–203 and DANA bound in active site between catalytic residues Glu-230/231, Tyr-342/343 and Asp59/60 in TcTS and TrSA respectively are displayed.

Among significant differences, the motif composed of amino acids 197–203 is IADMGGR in TrSA and Tr6, but is substituted to VTNKKKQ in TcTS. These substitutions cause a change in net charge of +3, and the MGGR → KKKQ sequence of substitution is highly unlikely to occur randomly (−4 in total from a BLOSUM62 matrix [22]). The substitution of two Gly residues to Lys could alter the rigidity of the loop. This changed rigidity might affect exclusion of water from the active site which could in turn explain the reduced hydrolytic activity observed in TcTS. To investigate this hypothesis, we compared the loops in structures of TcTS and TrSA with and without a ligand bound (Figure 2). A comparison of the loops shows little difference in the backbone position between the two enzymes and neither of them appeared to change conformation upon ligand binding. This indicates that the chemical properties of the side chains, rather than the size and backbone dynamics of the loops, are likely to determine differences in catalytic activity. Consequently, an alternative explanation for the role of the VTNKKKQ motif was sought in the introduction of a +3 charge difference between the two loops. This charge difference is also seen in the less similar trans-sialidase from *T. congolense* (ARNKANR, charge +3), but not in that of *T. brucei* (ARNEDNA, charge –1). We hypothesised that the charge difference and/or the substantial change towards hydrogen-bond donors could affect the affinity for lactose or reverse the water network in the substrate-binding cleft (or reduce water affinity, i.e. increase *K*_m_ for hydrolysis to favour competitive binding of lactose), all to the effect of reducing hydrolysis. Energetically, the free energy cost of aligning water molecules in the cleft (rearrangement free energy of ∼6.3 kJ/mol [23]) should be lower than for fully excluding them (free energy of hydration of water into water with an upper cost of ∼35 kJ/mol [24]).

**Figure 3:**
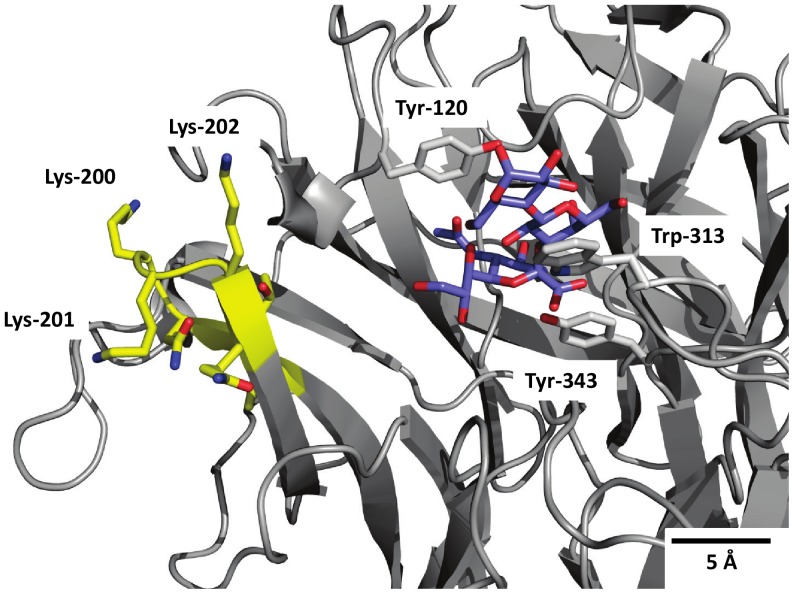
Homology model of Tr13. Close-up of active site with sialyllactose docked (blue). Acceptor binding site residues Tyr-120 and Trp-313 and catalytic nucleophile Tyr-343 side chains are shown in grey. The seven introduced amino acids are shown in yellow.

A homology model of mutant Tr13 based on Tr6 with the additional seven mutations of the VTNKKKQ motif is shown in Figure 3. The mutations are relatively far, ∼14 Å, from the acceptor binding site and therefore unlikely to affect acceptor binding directly. Water structure is from recent work known to be a combination of symmetric, directed tetrahedral structure and momentaneous asymmetry from breaking of hydrogen bonds [25]. The substantial increase of hydrogen bond donation from the loop side suggests that a tetrahedral water network of the symmetric type will be more likely to be inverted. This would change the electrostatic field in the cleft and potentially disrupt or even reverse the water network in the active site. Hydrolysis requires a water network aligned with oxygen lonepairs towards the sialic acid, whereas a strong positive charge and hydrogen-donor tendency at the edge of the binding cleft, as seen in the native trans-sialidase would work towards a partial reversal of such a network, turning oxygen lone pairs towards the field of the lysines and correspondingly impairing the nucleophilicity of the water network in the cleft. Such a disruption of the water network could be the explanation for TcTS's exquisite quenching of hydrolysis, not achieved by previous *T. rangeli* sialidase mutants [4].

In addition to the VTNKKKQ motif probed by Tr13, we also investigated several single-site mutations from Tr6, to understand the properties of previously described mutants in more detail. Two such mutations were reverting mutations of the parent, P98A and Y249G, while V180A was the mutant previously reported to at least partially reduce hydrolase activity [4]. As further candidates for improving trans-sialidase activity, several new single mutations were also made from Tr6: R125G, E175Q, G202K, N250R, Q123R, G127A, I177L, and D363E. These mutations were notable either by their positions close to the binding cleft and/or by conferring a chemical difference.

### Sialidase mutant Tr13 displays increased trans-sialylation activity

To assess the performance of the mutants, we produced them in *P. pastoris* shake flask cultures and tested their trans-sialidase activity using a fluorescence-based assay using cGMP as sialic acid donor and methylumbelliferyl-β-D-galactopyranoside (MU-Gal) as acceptor (Figure S2). The enzyme amounts obtained were too low to allow a detailed characterization of the mutants. Since the detected maximum product yield will be a measure of both the trans-sialidase activity (product formation) and the hydrolase activity (donor and product degradation) of the enzyme, it was considered a relevant assay for an initial screen of the mutants. Although all the mutants were active, only Tr13 and Tr6 D363E performed at a level comparable to Tr6 while all other mutants displayed a decreased trans-sialidase activity. These two mutants were therefore selected for further analysis, since the primary objective of this work was to generate mutants displaying improved trans-sialidase activity. It is worth noting that the single mutation G202K, which is part of the VTNKKKQ motif, reduced trans-sialidase activity. This indicates a co-operation effect of the amino acids of the motif and not an effect additively derived from the single-site mutations. For the reverting mutations P98A and Y249G it could be concluded that they improve but are not essential for trans-sialidase activity.

**Figure 4:**
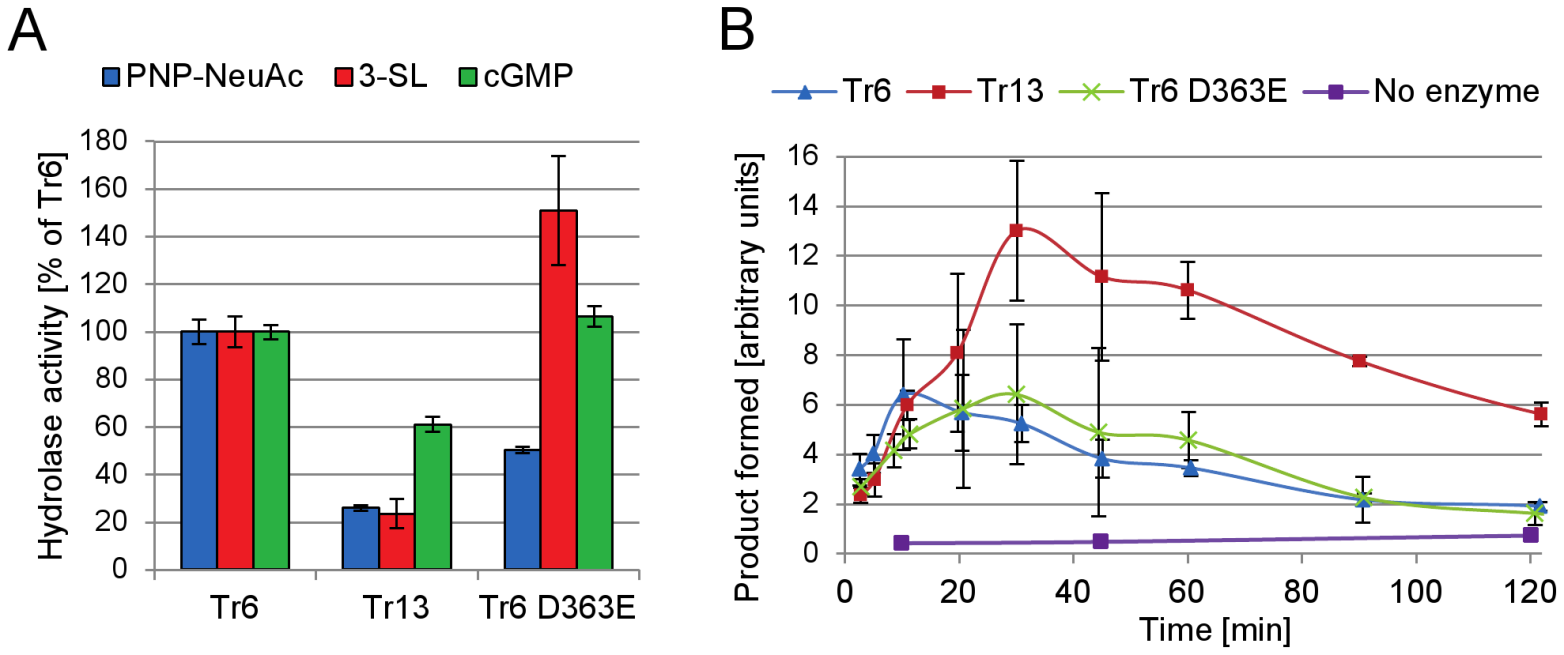
Enzyme activity of Tr6 and selected mutants Tr13 and Tr6 D363E. A) Hydrolase activity on substrates pNP-Neu5Ac, 3′-sialyllactose, and cGMP. B) Trans-sialidase activity using cGMP as sialic acid donor and MU-gal as acceptor.

For further evaluation we produced the parent enzyme Tr6 and the two mutants Tr13 and Tr6 D363E in a 5 L fermentor. This allowed us to evaluate both hydrolase and trans-sialidase activity (Figure 4). Hydrolase activity was tested on the artificial substrate pNP-Neu5Ac as well as on the natural substrates 3′-sialyllactose, 6′-sialyllactose, and cGMP. None of the enzymes exhibited detectable activity on 6′-sialyllactose (data not shown) in turn indicating that the α-2,6-linked sialic acid in cGMP was not used as a sialyl donor when cGMP was employed as donor substrate for the *T. rangeli* mutants. The α-2,6-linked sialic acid constitutes about 50% of total sialic acid content in cGMP [26]. While both Tr13 and Tr6 D363E had a decreased activity on pNP-Neu5Ac, only Tr13 had a lower hydrolase activity on 3′-sialyllactose and cGMP (Figure 4A).

In the trans-sialidase activity assay, the initial reaction rate represents the trans-sialidase reaction rate, while maximum product formation is a measure of both trans-sialidase activity (product formation) and hydrolase activity (product degradation). Thus, Tr6 and Tr13 appeared to have similar trans-sialidase activities (as measured by their initial reaction rates), while Tr6 D363E had a slightly lower activity, but under the applied reaction conditions Tr13 accomplished twice the maximal yield (Figure 4B), underscoring the importance of the VTNKKKQ motif for total yields due to reduced hydrolysis activity on the product.

The improved maximal yield obtained for Tr13 catalysis suggests that the VTNKKKQ motif does not affect the acceptor binding affinity, but rather uniquely reduces the hydrolytic activity (water *k*_cat_ and/or *K*_m_), probably by impairing water nucleophilicity for attack on sialic acid (partial reversal of the water network) and/or by reducing water's retention time in the active site in competition with the acceptor. The effect may be acceptor-dependent, as the total extent of hydrolysis not only depends on the impaired water network, but also the *K*_m_ of the acceptor during trans-sialylation, which affects acceptor *vs*. water retention time and thus, the competition between hydrolysis and trans-sialylation.

Of the three lysines introduced in Tr13, K200 and K201 are partly shielded from the active site (Figure 3) while K202 points towards the center of the site. Changing only K202 led to a reduced activity compared to the parent. The exact mechanism by which the mutations exert their effect remains unclear and certainly these mutations do not completely account for the exceptionally low hydrolase activity and the higher affinity for lactose in TcTS compared to Tr6. Tr13 however, represents a major step forward by providing the first-ever design of a markedly hydrolysis-impaired sialidase mutant. Within protein engineering at large, viable mutants with improved properties that deviate so substantially from a wild type (by 13 site changes including a 7-amino acid loop structure with a +3 charge difference) are unusual.

### Optimization of reaction conditions for Tr13

**Figure 5:**
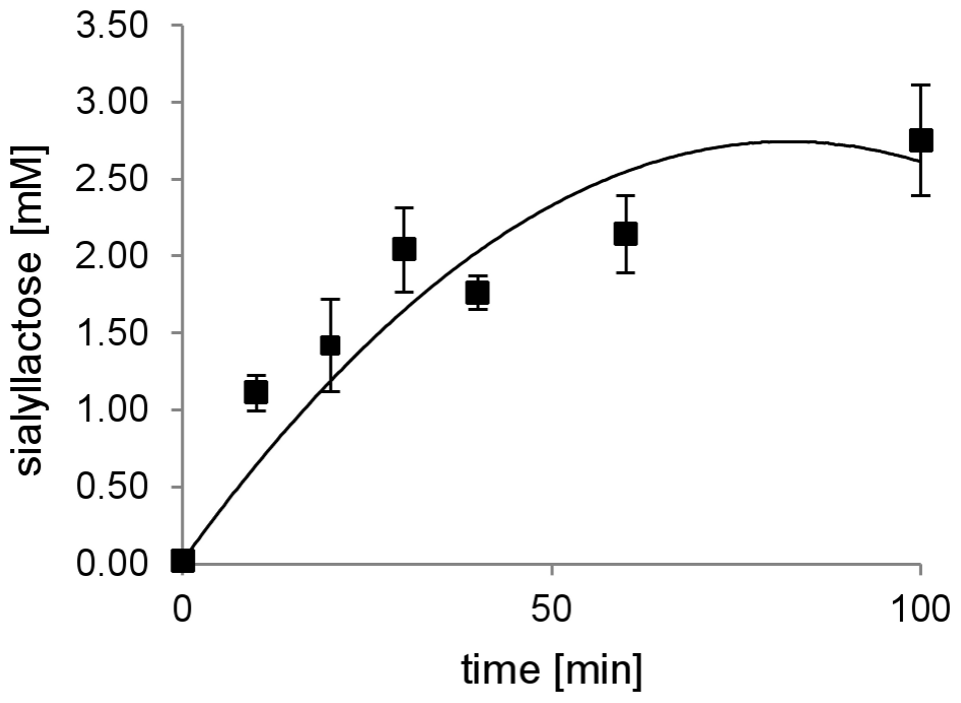
Time study of trans-sialylation catalysed by Tr13. Accumulation of 3′-sialyllactose over time in 25°C, pH 3, 351 mM lactose and 8 mM cGMP-bound sialic acid.

Statistically designed experiments were performed to determine reaction conditions that favour the trans-sialidase activity of Tr13. The influence of the following factors was investigated: temperature (20–60°C), pH (pH 3–5) and concentration of a standard acceptor for this reaction, lactose (117–351 mM), while using a fixed concentration of cGMP (8 mM). Reactions were allowed to proceed for 20 min, and after terminating the reactions, the concentration of 3′-sialyllactose was determined by HPAEC. No product formation was observed in controls with heat-inactivated enzyme. The highest product yield was obtained at 351 mM lactose (highest tested), pH 3 (lowest tested) and at 20°C (lowest tested) (data not shown). A time study was performed at these conditions and the specific trans-sialidase activity of the enzyme was determined from this study shown in Figure 5. The specific trans-sialidase activity, measured as number of sialyl-moieties transferred, of Tr13 was 4.4 +/−0.7 nmol*min^−1^ per µg of enzyme on cGMP. It was apparent that for a higher product yield, the reaction time could be successfully extended from 20 to 100 minutes with no detectable product degradation, since no free sialic acid was detected by LC/MS. Although the maximum yield was not determined in the study, it could be concluded that at least ∼2.5 mM 3′-sialyllactose could be produced. In cGMP, α-2, 3- and α-2,6-bound sialic acid is in a ratio of about 1∶1 [26], and hence only 4 of the 8 mM cGMP-bound sialic acid was theoretically accessible giving a yield of about 63%.

That the highest product yield was obtained at very low pH is surprising, since pH_opt_ for TcTS (using N-acetyllactosamine as acceptor) is pH 7 [27]. The introduction of charged residues in the vicinity of the catalytic residues might modulate the pH optimum but unlikely by such magnitude. A temperature optimum of 13°C has been reported for TcTS at low acceptor concentration (7.2 µM) due to increased affinity for the acceptor at low temperature while increasing acceptor concentration increased T_opt_ [28]. In our case, however, a very high acceptor concentration was used, indicating a different mechanism for the optimum at low temperature.

### Sialylation of various prebiotic glycans revealed promiscuous acceptor substrate specificity of Tr13

We also examined the possibility of using Tr13 for synthesis of chimeric molecules, probing the possibility of combining the prebiotic effect with sialic acid as found in many HMO molecules to assess potential, enhanced beneficial properties. To this end, we sialylated the well-documented prebiotics GOS, IMO, and lactulose as well as three other compounds, melibiose, maltose, and fucose. This also provided a possibility to probe the acceptor substrate specificity of this enzyme. For TcTS it is generally accepted that terminal galactose moieties function as acceptor with lactose being a better acceptor than melibiose and galactose, glucose being a poor one [29]. Here we tested terminal galactose, terminal glucose, and glucose and fucose monomers as acceptor substrates for Tr13.

**Figure 6:**
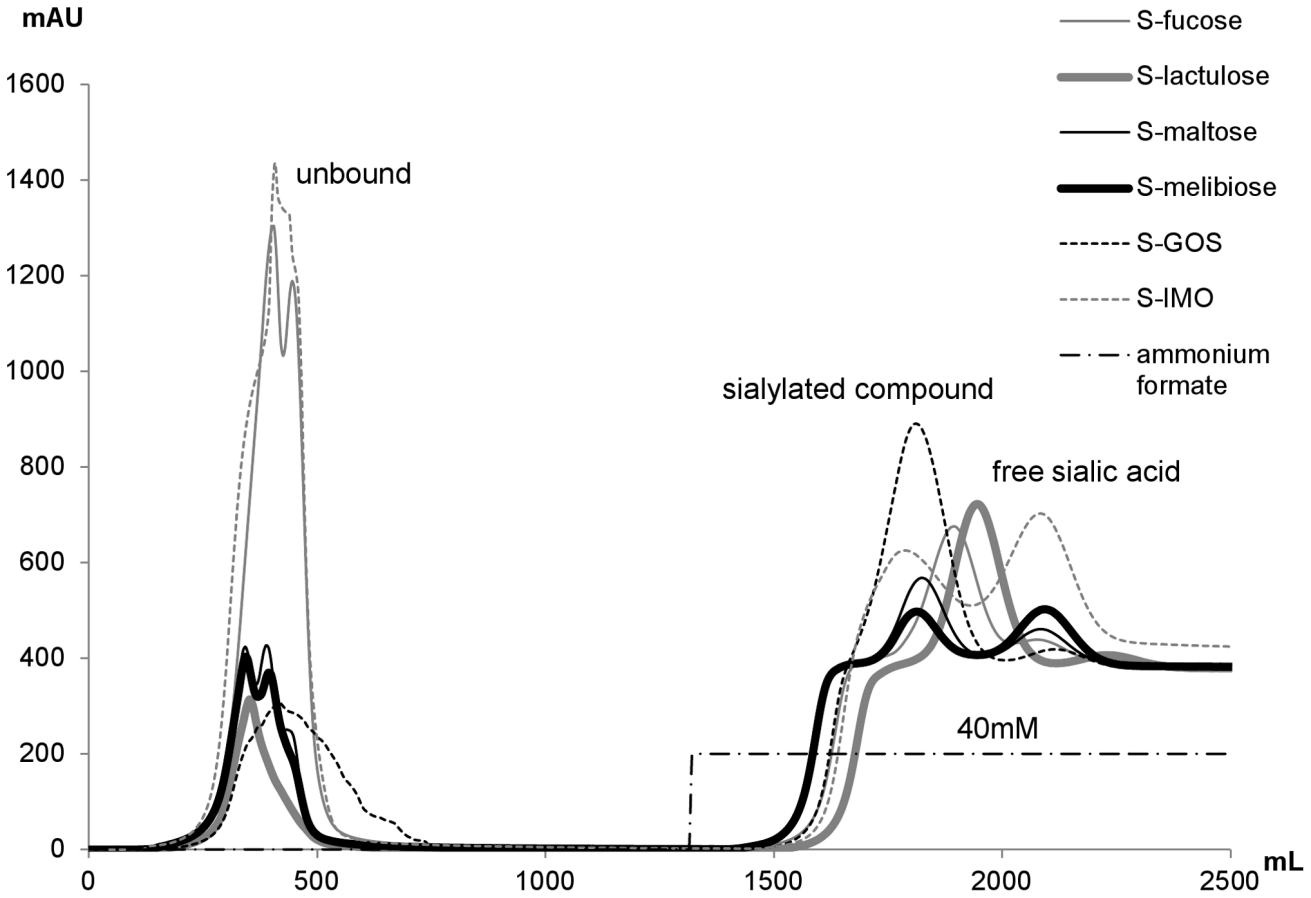
Anion exchange separation profiles for sialylated glycans. Sialylated glycans separated from sialic acid and unused acceptor separated by Sepharose Q and detected at 210

The sialylated oligosaccharides were enzymatically produced in phosphate-citrate buffer at pH 3 at ambient temperature. 351 mM sialic acid acceptor and 8 mM cGMP-bound sialic acid was used. After incubation for 20 minutes, the enzyme was heat-inactivated and the sialylated products were purified by anion exchange chromatography (Figure 6). In the unbound fractions, there was always neutral, unreacted acceptor, which was eluted with water. Applying 40 mM ammonium formate led to elution of negatively charged compounds, i.e. sialylated products and afterwards free sialic acid. According to LC/MS analysis, the sialylated compounds were completely separated from sialic acid as well as from the acceptor used in the reaction. The highest yields were achieved when fucose (1.5 mM) and lactulose (1.9 mM) were used as the sialic acid acceptors. Due to the relatively low yield compared to the concentration of acceptor substrates, it was relevant to assure that the substrates did not contain sialylated compounds. Analysis of the lactulose and melibiose preparations by LC/MS identified no contamination with sialylated compounds or sialic acid. The yields of sialylation products of GOS and IMO were lower. GOS and IMO preparations were a mixture of oligosaccharides of different chain length. The yields for each of the different chain lengths might have differed from the average since sialylation of smaller species appear to be favoured, at least for trans-sialylation with bacterial sialidases [30]. The composition of products of sialylation of GOS and IMO was complex (Table 2): Four and five different sialylated compounds, respectively, were obtained. In the case of GOS, the product of the lowest molecular weight was sialyllactose (m/z of 632), whereas incubation of IMO with cGMP led to production also of sialylated glucose (m/z of 470), since the starting material was abundant in that monomer.

**Table 2:** Products of sialylation of various glycans analysed by LC/MS.

acceptor	m/z	product	Product conc.	Product yield	
	[M-H]^−^		[g/L]	[w_prod_/w_accep_]	[mM]
GOS	632	SA-α-Gal-1,4-β-Glc	1.0	0.44%	ND
	794	SA-α-Gal-1,4-β-Gal-1,4-β-Glc			
	956	SA-α-Gal-1,4-β-Gal-1,4-β-Gal-1,4-β-Glc			
	1118	SA-α-Gal-1,4-β-Gal-1,4-β-Gal-1,4-β-Gal-1,4-β-Glc			
Fucose	454;	SA-α-Fuc	0.66	1.17%	1.46
Melibiose	632	SA-α-Gal-1,6-α-Glc	0.62	0.52%	0.98
Lactulose	632	SA-α-Gal-1,4-β-Fru	1.2	0.97%	1.84
Maltose	632	SA-α-Glc-1,4-α-Glc	0.66	0.55%	1.04
IMO	470	SA-α-Glc	0.72	0.60%	ND
	632	SA-α-Glc-α-Glc			
	794	SA-α-Glc-α-Glc-α-Glc			
	956	SA-α-Glc-α-Glc-α-Glc-α-Glc			
	1118	SA-α-Glc-α-Glc-α-Glc-α-Glc-α-Glc			

Yields are given as product concentration and as% (w/w) of product produced from acceptor used. ND; the molar concentration of sialylated GOS and IMO could not be calculated since the distribution of different chain lengths was not determined. Abbreviations: SA, sialic acid; Gal, galactose; Glc, glucose; Fuc, fucose; Fru, fructose.

Of the compounds produced, sialyllactulose was produced in the highest molar yield. The presence of galactose and the 1,4-β bond between galactose and fructose may make lactulose more accessible for Tr13, since for the similar-size acceptors melibiose (1,6-α-bound galactose) and maltose (1,4-α-bound glucose), yields were less than 60% of those obtained with lactulose. It is well established that glycans containing terminal galactose can be sialylated by TcTS, but the fact that Tr13 could also sialylate terminal glucose and even glucose and fucose monomers was surprising and indicated a high level of acceptor promiscuity. To our knowledge, this is the first report of such broad acceptor substrate specificity, and it can potentially be a valuable asset for enzymatic synthesis of a broad range of sialylated glycans using the Tr13 enzyme as catalyst.

### Potential prebiotic effect of various sialylated glycans

The end-goal for this ongoing research effort is the enzymatic synthesis of novel functional food ingredients. As an initial assessment of potential prebiotic properties of the sialylated glycans, their growth promoting activity on selected bacterial pure cultures comprising three strains of *B. longum* subs. *infantis*, two *B. longum* subsp. *longum*, one *B. lactis*, one *L. acidophilus* and one pathogenic strain of *C. perfringens* was evaluated. This was done using a Bioscreen microtiter system, and the growth performance was expressed as area under the growth curve [21]. To assess the impact of sialylation it would have been relevant to compare with growth on the unsialylated acceptor molecules, but since the distribution of sialylated molecules of different chain length in case of GOS and IMO was not quantified, we decided to use galactan from potato as a control due to its confirmed prebiotic properties [31].

The data presented in Table 3 revealed that within the group of probiotic strains, sialylated melibiose and maltose did not appear to promote growth. For *B. infantis* 233, *B. infantis* 1497, and *B. longum* 232, growth was promoted by different sialylated compounds while sialylated fucose promoted growth for all three. In the case of *B. infantis* 2238, *B. lactis*, *L. acidophilus*, and *B. longum* 9917, none of the sialylated compounds promoted growth, while *L. acidophilus* grew well on the prebiotic control substrate galactan. *C. perfringens* grew significantly better than all the probiotic strains on the sialylated compounds, except on sialylmelibiose where growth was not tested due to only a limited amount of material being available.

**Table 3:** Bacterial growth on sialylated glycans.

Bacterial strain	Area under the growth curve [OD_600_ x min]							
	MRS-	S-GOS	S-fucose	S-melibiose	S-lactulose	S-maltose	S-IMO	galactan
*B. infantis 233*	30±14	132±5	71±6	14±6	109±20	30±4	95±23	55±7
*B. infantis 2238*	294±68	274±18	285±15	302±2	269±20	158±24	269±6	264±7
*B. infantis 1497*	31±10	42±13	149±2	ND	34±2	ND	45±1	40±9
*B. longum 232*	79±20	162±9	192±20	104±17	134±19	122±31	107±19	42±13
*B. lactis*	139±70	176±18	192±27	ND	122±18	102±8	175±13	143±15
*L. acidophilus*	180±28	159±4	188±18	192±4	128±2	193±12	217±19	371±10
*B. longum 9917*	106±30	71±5	114±15	70±8	34±14	101±6	103±9	93±44
*C. perfringens*	455±32	722±52	811±48	ND	541±17	844±99	1098±61	447±46

Area under the growth curve for growth of probiotic strains and pathogenic *Clostridium perfringens* on sialylated glycans; MRS- represents growth in the medium with no carbohydrate added; galactan, an established prebiotic, was used as a positive control; growth responses for the substrates are shown for a substrate concentration of 10 g/L for all bacterial strains. Data are given as average values of 3 replicates and shown ± s.d. The growth of *B. longum infantis* 1497, *B. lactis* and *C. perfringens* was not tested on sialylmelibiose, as well as, growth of *B. longum infantis* 1497 on sialylmaltose (ND).

It was assumed that a prerequisite for utilising the sialylated compounds would be the presence of a sialidase, as well as the ability to degrade the prebiotic backbone. All the *B. longum subsp. infantis* strains contained a sialidase (data not shown) as does *C. perfringens*, which contains the necessary enzymes for metabolising sialic acid [32]. Although variations in growth were found on different substrates, even within species, it was evident that the majority of the bacteria tested, including *C. perfringens*, to some extent were able to grow on the sialylated compounds. Recently, three fucosylated HMOs were shown to stimulate bifidobacteria, while *E. coli* and *C. perfringens* were unable to utilise the HMOs [33]. More interestingly, the organic acid fermentation product inhibited their growth, raising the possibility that this might also take place in a mixed culture with the molecules synthesised in this study. A mixed culture experiment taking into account bacterial interactions would be required to assess this. Furthermore, the potential functionality of these compounds as decoy molecules and in modulation of the immune system will need to be addressed in future work.

## Concluding Remarks

Previous work by Paris and co-workers [4] identified important residues in the acceptor binding site and in the sialic acid-binding pocket that conferred trans-sialidase activity to the strict sialidase of *T. rangeli*. However, these mutants all retained a much higher (at least 100-fold) hydrolase activity compared to the native trans-sialidase of *T. cruzi*, indicating that a major determinant of impaired hydrolysis in the evolution of trans-sialidase was missing.

In this study, we have shown that a major chemical and structural difference between the native trans-sialidase TcTS and the homologous sialidase TrSA lies in the presence of a +3 charged VTNKKKQ motif on the edge of the acceptor binding cleft, and that this motif effectively reduces hydrolysis to promote trans-sialidase activity. The most likely function of this motif is to effectively disrupt (perhaps even reverse) the water binding network, which must be aligned with the oxygen lone pairs towards sialic acid in order to accomplish hydrolysis. Since the motif is not conserved among characterized trans-sialidases its beneficial effect might be context-dependent. Further structural and enzymatic studies will certainly be needed to assess the validity and generality of the proposed mechanisms.

A mutant, Tr13, was constructed from the parent Tr6 mutant, with this identified motif incorporated and it was shown to display a 4-fold lower hydrolase activity on 3′-sialyllactose, while trans-sialidase activity was essentially unaffected. As a result, a net doubling of the yield of sialylated product was obtained using this particular mutant Tr13, which also produced a better yield than a series of controlling single-site mutants. The mutant is therefore the first in a new class of envisioned mutants capable of reducing hydrolysis in competition with trans-sialidase activity by disruption of the water network of the acceptor-binding site and active site. Tr13 is also remarkable by being chemically substantially different from its wild type, with 13 sites mutated and a charge difference of +3.

Furthermore, the native TcTS is generally believed to use only terminal galactose as acceptor substrates [29], but Tr13 possessed an unusually broad acceptor-substrate specificity, accommodating terminal galactose but also terminal glucose and even monomers of glucose and fucose. This, together with the substantially reduced hydrolysis, should make Tr13 and its downstream mutants useful for enzymatic sialylation of a broad range of glycans in the pursuit of novel, functional food ingredients. To justify this approach, a number of established and potential prebiotics, including GOS, IMO, and lactulose, were sialylated in reasonable yields. An initial examination of prebiotic potential demonstrated growth of Bifidobacteria, although not selectively. Using Tr13 under optimal conditions should further allow a synthesis-scale of these novel molecules enabling further functionality tests including their prebiotic effect in mixed culture, and their potential as anti-adhesive antimicrobial and modulator of the immune system.

## Supporting Information

Figure S1 **The gene sequence and primary structure of Tr6.** Restriction sites used for vector construction are underlined. pPICZαC (Invitrogen)-encoded N-terminus containing α-factor signal sequence and Kex2 and Ste3 protease recognition sequence (amino acids 1–89) and C-terminus containing *c-myc* and 6xHis-tag (amino acids 730–752) in grey. Tr6 gene product (amino acids 90–729) in bold.
(PDF)

Figure S2 **Trans-sialidase activity of Tr6 and derived mutants.** Trans-sialidase activity measured using cGMP as sialic acid donor and methylumbellferyl-pyrogalactoside as acceptor. Product formation, for each of the mutant variants shown against that of the parent Tr6, is shown in arbitrary units. Differences in initial reaction rate might in part relate to differences in enzyme amount used.
(PDF)
