# Supplementary material for: Rational Design of a New Trypanosoma rangeli Trans-Sialidase for Efficient Sialylation of Glycans
Source: PLoS One. 2014 Jan 3;9(1):e83902. doi: 10.1371/journal.pone.0083902 (PMC3880268; doi:10.1371/journal.pone.0083902)
Supplement: Figure S1 — The gene sequence and primary structure of Tr6. Restriction sites used for vector construction are underlined. pPICZαC (Invitrogen)-encoded N-terminus containing α-factor signal sequence and Kex2 and Ste3 protease recognition sequence (amino acids 1–89) and C-terminus containing c-myc and 6xHis-tag (amino acids 730–752) in grey. Tr6 gene product (amino acids 90–729) in bold. (PDF) [file pone.0083902.s001.pdf]

1 atgagatttccttcaatttttactgctgttttattcgcagcatcctccgcattagctgct

M R F P S I F T A V L F A A S S A L A A

60 ccagtcaacactacaacagaagatgaaacggcacaaattccggctgaagctgtcatcggt

P V N T T T E D E T A Q I P A E A V I G

120 tactcagatttagaaggggatttcgatgttgctgttttgccattttccaacagcacaaat

Y S D L E G D F D V A V L P F S N S T N

180 aacgggttattgtttataaatactactattgccagcattgctgctaaagaagaaggggta

N G L L F I N T T I A S I A A K E E G V

*Xho*I

240 tctctcgagaagagagaggctgaagctgcttctttggctcccggatcatctcgtgtggaa

S L E K R E A E A **A S L A P G S S R V E**

300 ttatttaaaagaaaaaactccaccgtgccatttgaggagtcaaacggtactatacgagaa

L F K R K N S T V P F E E S N G T I R E

360 cgtgtggttcattcatttagattaccaactatcgttaacgtagatggagtcatggttgcc

R V V H S F R L P T I V N V D G V M V A

420 attgctgatgccagatatgagacatcattcgacaactcctttatcgaaactgctgttaaa

I A D A R Y E T S F D N S F I E T A V K

480 tacagtgttgacgatggtgctacgtggaatacacaaattgcaatcaaaaattctcgtgca

Y S V D D G A T W N T Q I A I K N S R A

540 tcatcagtttcaagggttgtcgatcctacggtcatagtaaagggaaataagttgtatatc

S S V S R V V D P T V I V K G N K L Y I

600 ctggttggatcctttaacaagacaaggaactattggacccagcacagagatggatctgac

L V G S F N K T R N Y W T Q H R D G S D

660 tgggaaccattgttggtggttggagaggttacgaagtctgctgctaacggtaaaacaact

W E P L L V V G E V T K S A A N G K T T

720 gcaactatttcatggggaaaacctgtctcccttaagcctttgttccctgcagagttcgac

A T I S W G K P V S L K P L F P A E F D

780 ggcatacttactaaggaattcgtaggtggagtaggcgccgccatcgtggcaagtaatggt

G I L T K E F V G G V G A A I V A S N G

840 aatttggtataccctgtgcaaattgctgacatgggaggaagagtatttacaaaaattatg

N L V Y P V Q I A D M G G R V F T K I M

900 tattccgaggatgatggtaacacttggaagttcgccgaaggaaggtctaagttcggttgc

Y S E D D G N T W K F A E G R S K F G C

960 tcagaaccagcagttttggaatgggaaggaaagctaatcattaataaccgagtcgattac

S E P A V L E W E G K L I I N N R V D Y

1020 aatagacgtctggtgtacgaatccagtgacatgggcaaaacatgggtagaggctcttggt

N R R L V Y E S S D M G K T W V E A L G

1080 actctgtcccacgtctggacgaacagtccaacttccaatcaacccgattgtcagagttca

T L S H V W T N S P T S N Q P D C Q S S

1140 ttcgttgcagttactatcgaaggtaaacgagtgatgttgtttactcatccactaaatttg

F V A V T I E G K R V M L F T H P L N L

1200 aagggtagatggatgagggatagacttcatctgtggatgaccgataatcagagaatcttt
K G R W M R D R L H L W M T D N Q R I F

1260 gatgttggccaaatttccattggtgatgaaaacagtggttactcttccgtcctatacaag
D V G Q I S I G D E N S G Y S S V L Y K

1320 gacgataaattatattccctacatgagattaatactaatgatgtttattctcttgttttt
D D K L Y S L H E I N T N D V Y S L V F

1380 gtccgattgattggtgagctgcagttaatgaaaagtgtggttcgtacctggaaggaagag
V R L I G E L Q L M K S V V R T W K E E

1440 gacaatcatttggcttcaatatgtactccagtcgtaccagcaaccccaccaagtaaagga
D N H L A S I C T P V V P A T P P S K G

1500 gcctgcggtgccgctgtacctacagctggtttagttggcttcttatctcactcagctaat
A C G A A V P T A G L V G F L S H S A N

1560 ggatccgtttgggaggacgtatatagatgtgtcgatgctaacgtcgccaacgctgagaga
G S V W E D V Y R C V D A N V A N A E R

1620 gttcctaacggccttaagtttaatggggttggtgggggcgctgtctggccagtcgccagg
V P N G L K F N G V G G G A V W P V A R

1680 cagggacaaacccgaaggtaccaattcgcaaactacagatttaccttagtcgccaccgtt
Q G Q T R R Y Q F A N Y R F T L V A T V

1740 acgattgacgaattgcccaaaggtacctctccccttcttggtgccgggttagaaggtcca
T I D E L P K G T S P L L G A G L E G P

1800 ggcgacgctaaattgctaggtttatcttacgacaagaaccgtcaatggcgacccttgtac
G D A K L L G L S Y D K N R Q W R P L Y

1860 ggagcagcccctgcttctcctacaggatcttgggagctacacaagaagtaccatgtagtc
G A A P A S P T G S W E L H K K Y H V V

1920 ctgaccatggctgacagacaggggagtgtttatgttgatgggcaacctcttgccggatca
L T M A D R Q G S V Y V D G Q P L A G S

1980 ggcaataccgtggttagaggagctactttgccagacatctctcacttctacattggtgga
G N T V V R G A T L P D I S H F Y I G G

2040 cccagatctaagggagcccctacagatagtagagttacagtgactaacgtggttttatat
P R S K G A P T D S R V T V T N V V L Y

2100 aatcgtagactgaactctagtgagatccgtacactttttttgtctcaagacatgatagg t
N R R L N S S E I R T L F L S Q D M I G

*Xba*I

2160 accgatggtggagctggtacagcagcatttctagaacaaaaactcatctcagaagaggat
T D G G A G T A A F L E Q K L I S E E D

2220 ctgaatagcgccgtcgaccatcatcatcatcatcattga
L N S A V D H H H H H H -
