# Supplementary figures and images for: Rational Design of a New Trypanosoma rangeli Trans-Sialidase for Efficient Sialylation of Glycans
Source: PLoS One. 2014 Jan 3;9(1):e83902. doi: 10.1371/journal.pone.0083902 (PMC3880268; doi:10.1371/journal.pone.0083902)

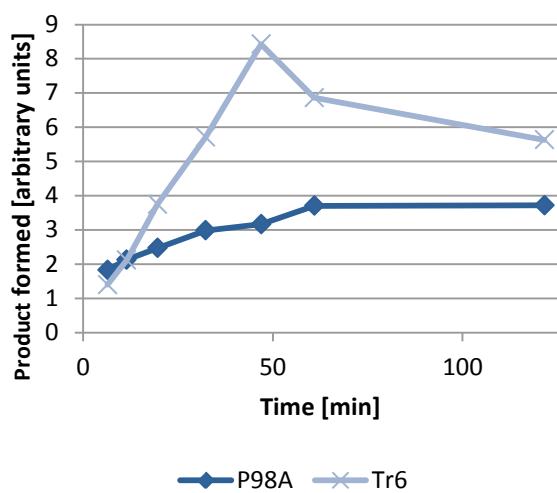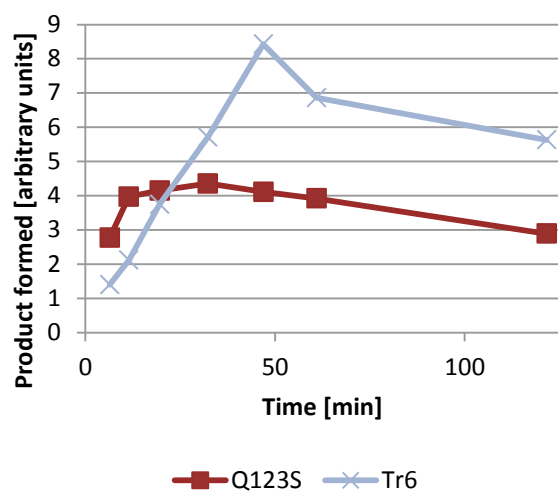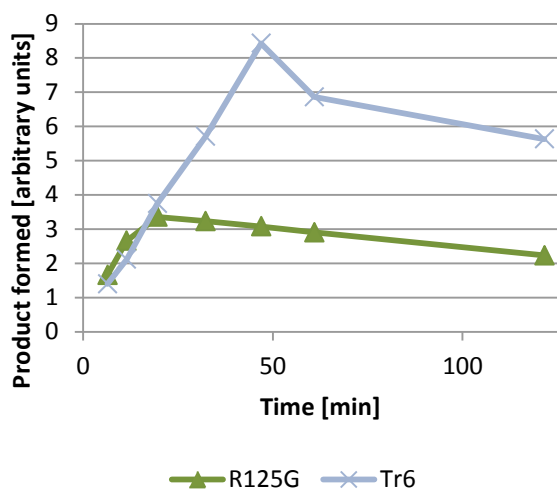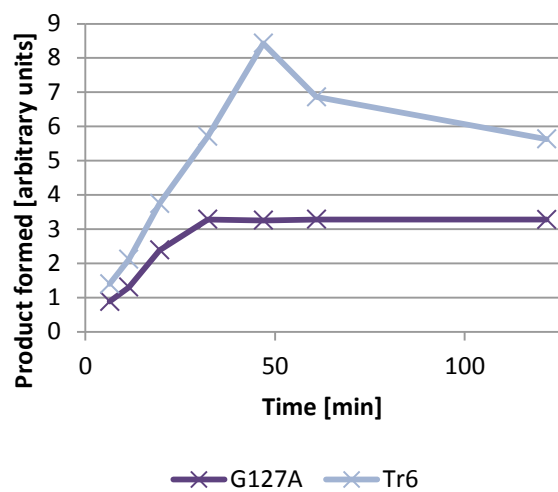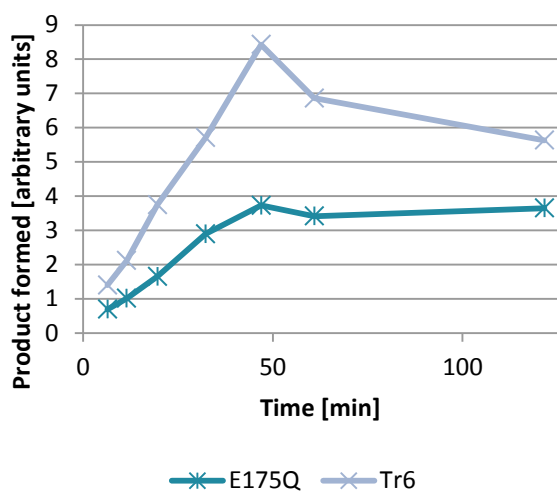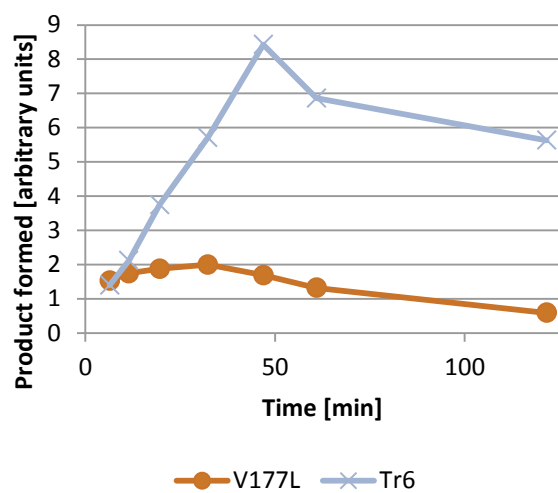

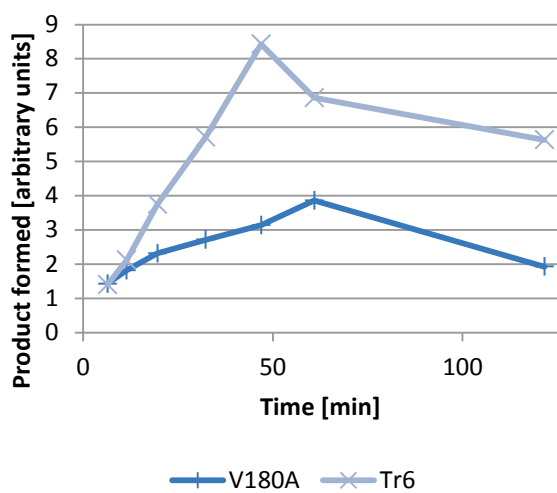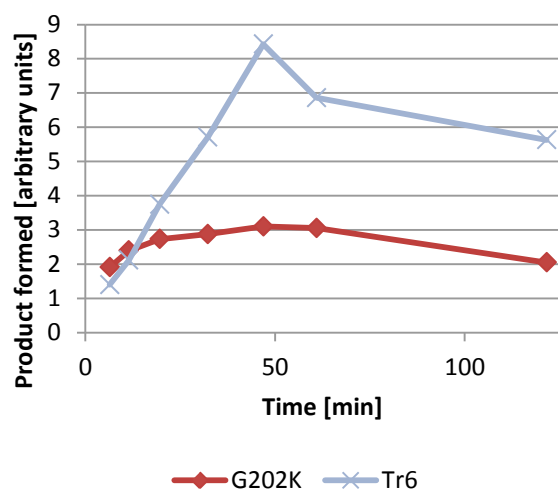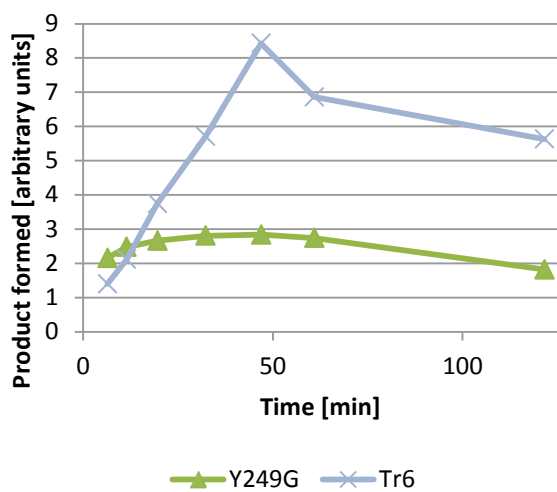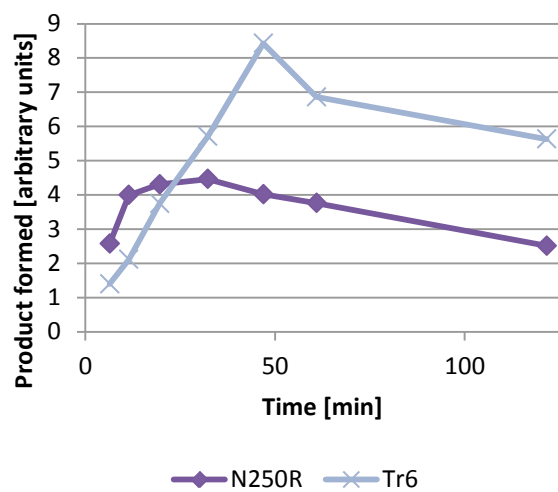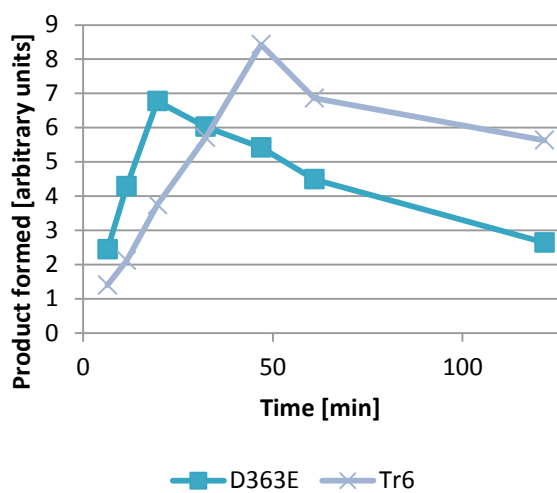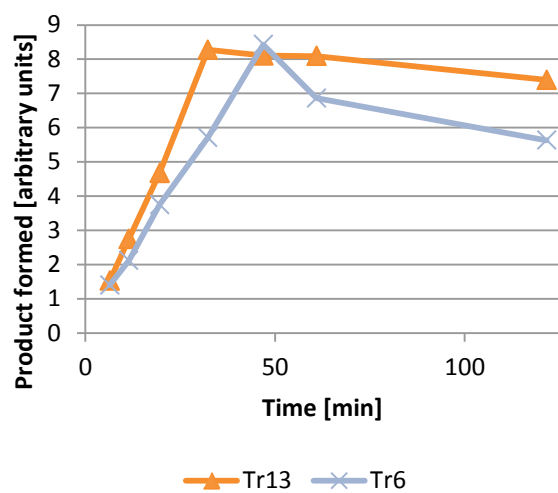

Supplement: Figure S2 — Trans-sialidase activity of Tr6 and derived mutants. Trans-sialidase activity measured using cGMP as sialic acid donor and methylumbellferyl-pyrogalactoside as acceptor. Product formation, for each of the mutant variants shown against that of the parent Tr6, is shown in arbitrary units. Differences in initial reaction rate might in part relate to differences in enzyme amount used. (PDF) [file pone.0083902.s002.pdf]
